# 3-Acetyl-5-hy­droxy-2-methyl­anthra[1,2-*b*]furan-6,11-dione

**DOI:** 10.1107/S1600536811013389

**Published:** 2011-04-16

**Authors:** Rohaya Ahmad, Mohamad Faiz Jeinie, Nor Hadiani Ismail, Hazrina Hazni, Seik Weng Ng

**Affiliations:** a Faculty of Applied Sciences, Universiti Teknologi MARA, Shah Alam 40450, Selangor, Malaysia; b Department of Chemistry, University of Malaya, 50603 Kuala Lumpur, Malaysia

## Abstract

The asymmetric unit of the title compound, C_19_H_12_O_5_, contains two independent mol­ecules, both slightly buckled along an axis passing through the C=O bonds of the anthraquinone ring system (r.m.s. deviation of non-H atoms = 0.082 and 0.148 Å): the benzene rings are twisted to each other by 4.3 (3)°in one mol­ecule and 10.6(3)° in the other. In both mol­ecules, the hy­droxy group forms an intra­molecular O—H⋯O hydrogen bond. The two independent mol­ecules inter­act by π–π stacking with a centroid–centroid distance of 3.539 (2) Å between hy­droxy­benzene rings of adjacent mol­ecules.

## Related literature

For background to the synthesis, see: Boddy *et al.* (1986 ▶).
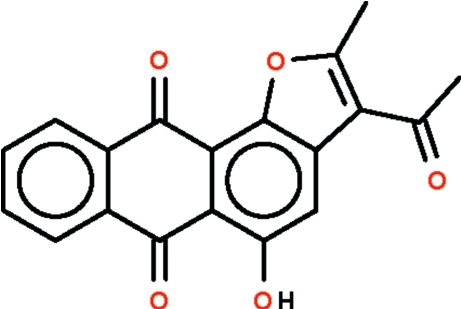


## Experimental

### 

#### Crystal data

C_19_H_12_O_5_
*M* *_r_* = 320.29
Monoclinic, 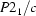

*a* = 12.5739 (9) Å
*b* = 22.0375 (12) Å
*c* = 10.7453 (8) Å
β = 110.342 (8)°
*V* = 2791.8 (3) Å^3^
*Z* = 8
Mo *K*α radiation
μ = 0.11 mm^−1^
*T* = 100 K
0.35 × 0.05 × 0.02 mm

#### Data collection

Agilent SuperNova Dual diffractometer with an Atlas detector
Absorption correction: multi-scan (*CrysAlis PRO*; Agilent, 2010 ▶) *T*_min_ = 0.962, *T*_max_ = 0.998
12061 measured reflections
4905 independent reflections
2305 reflections with *I* > 2σ(*I*)
*R*_int_ = 0.093

#### Refinement

*R*[*F* ^2^ > 2σ(*F* ^2^)] = 0.068
*wR*(*F* ^2^) = 0.149
*S* = 0.95
4905 reflections
445 parameters
2 restraints
H atoms treated by a mixture of independent and constrained refinement
Δρ_max_ = 0.25 e Å^−3^
Δρ_min_ = −0.28 e Å^−3^

### 

Data collection: *CrysAlis PRO* (Agilent, 2010 ▶); cell refinement: *CrysAlis PRO*; data reduction: *CrysAlis PRO*; program(s) used to solve structure: *SHELXS97* (Sheldrick, 2008 ▶); program(s) used to refine structure: *SHELXL97* (Sheldrick, 2008 ▶); molecular graphics: *X-SEED* (Barbour, 2001 ▶); software used to prepare material for publication: *publCIF* (Westrip, 2010 ▶).

## Supplementary Material

Crystal structure: contains datablocks global, I. DOI: 10.1107/S1600536811013389/xu5188sup1.cif

Structure factors: contains datablocks I. DOI: 10.1107/S1600536811013389/xu5188Isup2.hkl

Additional supplementary materials:  crystallographic information; 3D view; checkCIF report

## Figures and Tables

**Table 1:** Hydrogen-bond geometry (Å, °)

*D*—H⋯*A*	*D*—H	H⋯*A*	*D*⋯*A*	*D*—H⋯*A*
O5—H5⋯O4	0.84 (1)	1.82 (2)	2.596 (4)	153 (4)
O10—H10⋯O9	0.84 (1)	1.77 (3)	2.552 (4)	153 (6)

**Fig. 1.**
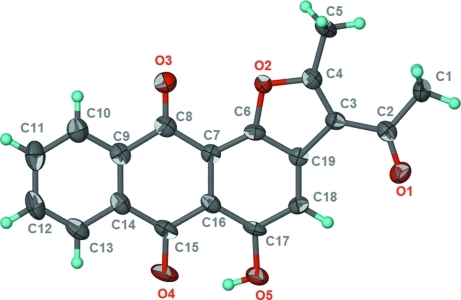
Thermal ellipsoid plot (Barbour, 2001) of one independent molecule of C19H12O5 at the 70% probability level; hydrogen atoms are drawn as spheres of arbitrary radius.

**Fig. 2.**
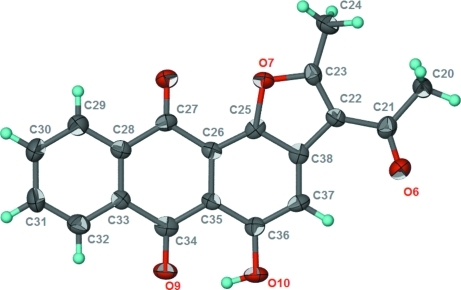
Thermal ellipsoid plot (Barbour, 2001) of second independent molecule of C19H12O5 at the 70% probability level; hydrogen atoms are drawn as spheres of arbitrary radius.

**Fig. 3.**
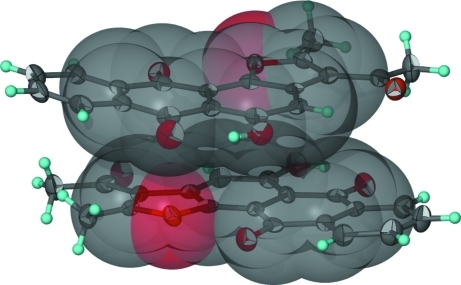
π–π Stacking of the two independent molecules of C19H12O5.

## supplementary crystallographic information

### Comment

The title compound (Scheme I) is one of the intermediates of the multi-stepsynthesis of a substituted isobenzofuran from anthradifuran (Boddy *et al.*, 1986). In the present microwave-assisted synthesis, even the 1,4-dihdyroanthraquinone reactant can be prepared under microwave conditions.

The two independent molecules C_19_H_12_O_5_ (Fig. 1 and Fig. 2) are both slightly buckled along an axis passing through the C═O bonds of the anthraquinone ring-system; for one molecule, the benzene rings are twisted by 4.3 (3) ° and for the other, the benzene rings are twisted by 10.6 (3)°. The two independent molecules interact by π–π stacking with a centroid-to-centroid distance of 3.539 (2) Å between hydroxybenzene rings of adjacent molecules.

### Experimental

Phthalic anhydride (0.001 mol) and hydroquinone (0.001 mol) were reacted over montmorillonite K10 clay (without any solvent) in a microwave oven. The reactants were subject to microwave radiation for 1 h. This afforded 1,4-dihydroxyanthraquinone in 95% yield. In the next step, acetylacetone (0.001 mol) was added to the prepared 1,4-dihydroxyanthraquinone (0.001 mol) in the presence of 4-dimethylaminopyridine (0.01 mol). The mixture was agained subject to microwave irradiation (100% power level for 1 h) to give the title compound, whose formulation was established by 1H NMR spectroscopy; yield 95% yield. Orange crystals were obtained by recrystallization from a hexane-chloroform (5:95) mixture.

### Refinement

Carbon-bound H-atoms were placed in calculated positions [C—H 0.95 to 0.98 Å, *U*_iso_(H) 1.2 to 1.5*U*_eq_(C)] and were included in the refinement in the riding model approximation.

The hydroxy H-atoms were located in a difference Fourier map, and were refined with distance restraints of O–H 0.84±0.01 Å; their temperature factors were refined.

### Crystal data

**Table:**

C_19_H_12_O_5_	*F*(000) = 1328
*M**_r_* = 320.29	*D*_x_ = 1.524 Mg m^−^^3^
Monoclinic, *P*2_1_/*c*	Mo *K*α radiation, λ = 0.71073 Å
Hall symbol: -P 2ybc	Cell parameters from 1530 reflections
*a* = 12.5739 (9) Å	θ = 2.2–29.1°
*b* = 22.0375 (12) Å	µ = 0.11 mm^−^^1^
*c* = 10.7453 (8) Å	*T* = 100 K
β = 110.342 (8)°	Plate, orange
*V* = 2791.8 (3) Å^3^	0.35 × 0.05 × 0.02 mm
*Z* = 8	

### Data collection

**Table:**

Agilent SuperNova Dual diffractometer with an Atlas detector	4905 independent reflections
Radiation source: SuperNova (Mo) X-ray Source	2305 reflections with *I* > 2σ(*I*)
Mirror	*R*_int_ = 0.093
Detector resolution: 10.4041 pixels mm^-1^	θ_max_ = 25.0°, θ_min_ = 2.2°
ω scans	*h* = −14→14
Absorption correction: multi-scan (*CrysAlis PRO*; Agilent, 2010)	*k* = −26→21
*T*_min_ = 0.962, *T*_max_ = 0.998	*l* = −12→9
12061 measured reflections	

### Refinement

**Table:**

Refinement on *F*^2^	Primary atom site location: structure-invariant direct methods
Least-squares matrix: full	Secondary atom site location: difference Fourier map
*R*[*F*^2^ > 2σ(*F*^2^)] = 0.068	Hydrogen site location: inferred from neighbouring sites
*wR*(*F*^2^) = 0.149	H atoms treated by a mixture of independent and constrained refinement
*S* = 0.95	*w* = 1/[σ^2^(*F*_o_^2^) + (0.029*P*)^2^] where *P* = (*F*_o_^2^ + 2*F*_c_^2^)/3
4905 reflections	(Δ/σ)_max_ = 0.001
445 parameters	Δρ_max_ = 0.25 e Å^−^^3^
2 restraints	Δρ_min_ = −0.28 e Å^−^^3^

### Fractional atomic coordinates and isotropic or equivalent isotropic displacement parameters (Å^2^)

**Table:**

	*x*	*y*	*z*	*U*_iso_*/*U*_eq_	
O1	0.1639 (2)	0.63359 (13)	0.4656 (3)	0.0307 (8)	
O2	0.1956 (2)	0.42672 (12)	0.4828 (3)	0.0225 (7)	
O3	0.1802 (2)	0.32131 (13)	0.6137 (3)	0.0308 (8)	
O4	0.0110 (2)	0.44812 (14)	0.9299 (3)	0.0406 (9)	
O5	0.0397 (3)	0.55000 (14)	0.8246 (3)	0.0303 (8)	
O6	0.4668 (2)	0.26821 (13)	0.8213 (3)	0.0361 (8)	
O7	0.3238 (2)	0.41683 (13)	1.0149 (3)	0.0256 (7)	
O8	0.2983 (2)	0.53832 (13)	1.0463 (3)	0.0315 (8)	
O9	0.4542 (2)	0.58374 (13)	0.6465 (3)	0.0297 (8)	
O10	0.4846 (3)	0.47096 (16)	0.6165 (3)	0.0313 (8)	
C1	0.2569 (4)	0.60289 (19)	0.3177 (5)	0.0344 (13)	
H1A	0.2554	0.6464	0.2986	0.052*	
H1B	0.2167	0.5807	0.2358	0.052*	
H1C	0.3357	0.5889	0.3537	0.052*	
C2	0.2001 (3)	0.5916 (2)	0.4175 (4)	0.0242 (11)	
C3	0.1909 (3)	0.52918 (19)	0.4607 (4)	0.0218 (11)	
C4	0.2152 (3)	0.4754 (2)	0.4141 (4)	0.0235 (11)	
C5	0.2569 (3)	0.4558 (2)	0.3062 (4)	0.0291 (11)	
H5A	0.3127	0.4232	0.3390	0.044*	
H5B	0.2925	0.4904	0.2784	0.044*	
H5C	0.1930	0.4411	0.2304	0.044*	
C6	0.1547 (3)	0.45001 (19)	0.5772 (4)	0.0205 (10)	
C7	0.1232 (3)	0.41721 (18)	0.6675 (4)	0.0195 (10)	
C8	0.1320 (3)	0.3503 (2)	0.6764 (4)	0.0249 (11)	
C9	0.0804 (3)	0.31883 (19)	0.7640 (4)	0.0240 (11)	
C10	0.0730 (3)	0.2559 (2)	0.7613 (4)	0.0302 (12)	
H10A	0.1046	0.2333	0.7073	0.036*	
C11	0.0198 (4)	0.2258 (2)	0.8367 (5)	0.0373 (13)	
H11	0.0144	0.1828	0.8339	0.045*	
C12	−0.0259 (4)	0.2590 (2)	0.9169 (5)	0.0370 (13)	
H12	−0.0635	0.2385	0.9676	0.044*	
C13	−0.0167 (3)	0.3215 (2)	0.9230 (4)	0.0302 (12)	
H13	−0.0455	0.3438	0.9799	0.036*	
C14	0.0356 (3)	0.3520 (2)	0.8445 (4)	0.0249 (11)	
C15	0.0417 (3)	0.4196 (2)	0.8474 (4)	0.0280 (12)	
C16	0.0813 (3)	0.45079 (19)	0.7526 (4)	0.0219 (10)	
C17	0.0767 (3)	0.5151 (2)	0.7443 (4)	0.0223 (11)	
C18	0.1098 (3)	0.54647 (19)	0.6509 (4)	0.0210 (10)	
H18	0.1059	0.5895	0.6459	0.025*	
C19	0.1483 (3)	0.51364 (18)	0.5662 (4)	0.0178 (10)	
C20	0.3954 (4)	0.22087 (18)	0.9754 (4)	0.0335 (12)	
H20A	0.4295	0.1844	0.9528	0.050*	
H20B	0.3133	0.2150	0.9502	0.050*	
H20C	0.4284	0.2282	1.0712	0.050*	
C21	0.4183 (4)	0.2744 (2)	0.9022 (4)	0.0273 (11)	
C22	0.3851 (3)	0.33562 (18)	0.9271 (4)	0.0216 (10)	
C23	0.3357 (3)	0.35386 (19)	1.0167 (4)	0.0232 (11)	
C24	0.2934 (4)	0.32387 (19)	1.1114 (4)	0.0331 (12)	
H24A	0.2659	0.2832	1.0789	0.050*	
H24B	0.2310	0.3477	1.1215	0.050*	
H24C	0.3548	0.3206	1.1975	0.050*	
C25	0.3663 (3)	0.43878 (19)	0.9207 (4)	0.0211 (10)	
C26	0.3693 (3)	0.49820 (19)	0.8854 (4)	0.0188 (10)	
C27	0.3289 (3)	0.54833 (19)	0.9518 (4)	0.0223 (10)	
C28	0.3271 (3)	0.61072 (19)	0.8981 (4)	0.0223 (11)	
C29	0.2850 (4)	0.65801 (19)	0.9521 (5)	0.0303 (12)	
H29	0.2585	0.6506	1.0235	0.036*	
C30	0.2814 (4)	0.7162 (2)	0.9015 (5)	0.0341 (12)	
H30	0.2503	0.7485	0.9364	0.041*	
C31	0.3238 (4)	0.7273 (2)	0.7989 (5)	0.0346 (12)	
H31	0.3232	0.7673	0.7658	0.042*	
C32	0.3665 (3)	0.6800 (2)	0.7460 (5)	0.0309 (12)	
H32	0.3942	0.6874	0.6756	0.037*	
C33	0.3689 (3)	0.62214 (19)	0.7951 (4)	0.0203 (10)	
C34	0.4148 (3)	0.5720 (2)	0.7356 (4)	0.0249 (11)	
C35	0.4106 (3)	0.51037 (19)	0.7802 (4)	0.0193 (10)	
C36	0.4479 (3)	0.46163 (19)	0.7192 (4)	0.0216 (10)	
C37	0.4450 (3)	0.4014 (2)	0.7599 (4)	0.0246 (11)	
H37	0.4705	0.3692	0.7187	0.030*	
C38	0.4041 (3)	0.39022 (19)	0.8614 (4)	0.0218 (11)	
H5	0.022 (3)	0.5247 (15)	0.873 (4)	0.034 (15)*	
H10	0.478 (5)	0.5087 (7)	0.603 (6)	0.09 (2)*	

### Atomic displacement parameters (Å^2^)

**Table:**

	*U*^11^	*U*^22^	*U*^33^	*U*^12^	*U*^13^	*U*^23^
O1	0.0392 (19)	0.0233 (18)	0.030 (2)	−0.0006 (15)	0.0133 (16)	−0.0033 (16)
O2	0.0276 (17)	0.0213 (17)	0.0210 (18)	0.0004 (14)	0.0117 (14)	0.0014 (14)
O3	0.0354 (19)	0.0278 (19)	0.031 (2)	−0.0003 (15)	0.0138 (16)	−0.0033 (16)
O4	0.051 (2)	0.047 (2)	0.037 (2)	0.0000 (17)	0.0321 (19)	−0.0083 (18)
O5	0.0377 (19)	0.031 (2)	0.029 (2)	−0.0037 (17)	0.0201 (17)	−0.0064 (17)
O6	0.048 (2)	0.0312 (19)	0.036 (2)	0.0053 (17)	0.0242 (18)	−0.0004 (17)
O7	0.0329 (18)	0.0278 (19)	0.0185 (18)	0.0013 (15)	0.0118 (15)	0.0035 (15)
O8	0.045 (2)	0.0299 (19)	0.024 (2)	−0.0007 (16)	0.0181 (17)	−0.0013 (16)
O9	0.0312 (18)	0.036 (2)	0.028 (2)	0.0023 (15)	0.0180 (16)	0.0068 (16)
O10	0.0380 (19)	0.037 (2)	0.027 (2)	0.0013 (18)	0.0215 (16)	0.0040 (18)
C1	0.047 (3)	0.024 (3)	0.039 (3)	0.003 (2)	0.023 (3)	0.008 (2)
C2	0.022 (2)	0.029 (3)	0.020 (3)	−0.003 (2)	0.007 (2)	0.000 (2)
C3	0.020 (2)	0.025 (3)	0.020 (3)	−0.001 (2)	0.007 (2)	−0.003 (2)
C4	0.021 (2)	0.029 (3)	0.019 (3)	0.001 (2)	0.004 (2)	0.002 (2)
C5	0.033 (3)	0.033 (3)	0.029 (3)	−0.001 (2)	0.020 (2)	−0.001 (2)
C6	0.017 (2)	0.027 (3)	0.016 (3)	0.002 (2)	0.004 (2)	−0.004 (2)
C7	0.015 (2)	0.024 (3)	0.016 (3)	−0.002 (2)	0.0015 (19)	−0.002 (2)
C8	0.022 (2)	0.032 (3)	0.017 (3)	−0.001 (2)	0.003 (2)	0.000 (2)
C9	0.022 (2)	0.031 (3)	0.015 (3)	−0.002 (2)	0.001 (2)	0.001 (2)
C10	0.029 (3)	0.034 (3)	0.025 (3)	0.000 (2)	0.006 (2)	0.005 (2)
C11	0.042 (3)	0.036 (3)	0.029 (3)	−0.003 (3)	0.006 (3)	0.011 (3)
C12	0.036 (3)	0.046 (3)	0.028 (3)	−0.003 (3)	0.010 (2)	0.016 (3)
C13	0.029 (3)	0.044 (3)	0.020 (3)	−0.001 (2)	0.011 (2)	0.002 (2)
C14	0.020 (2)	0.033 (3)	0.019 (3)	−0.002 (2)	0.005 (2)	0.004 (2)
C15	0.025 (3)	0.039 (3)	0.024 (3)	−0.003 (2)	0.014 (2)	−0.004 (2)
C16	0.016 (2)	0.029 (3)	0.020 (3)	−0.002 (2)	0.007 (2)	0.000 (2)
C17	0.020 (2)	0.035 (3)	0.015 (3)	0.001 (2)	0.009 (2)	−0.006 (2)
C18	0.021 (2)	0.020 (2)	0.020 (3)	−0.002 (2)	0.005 (2)	−0.006 (2)
C19	0.010 (2)	0.023 (3)	0.015 (3)	−0.002 (2)	−0.0008 (19)	−0.002 (2)
C20	0.053 (3)	0.024 (3)	0.029 (3)	0.004 (2)	0.020 (3)	0.003 (2)
C21	0.031 (3)	0.025 (3)	0.023 (3)	−0.002 (2)	0.006 (2)	−0.007 (2)
C22	0.022 (2)	0.023 (3)	0.018 (3)	−0.004 (2)	0.005 (2)	0.000 (2)
C23	0.025 (2)	0.018 (3)	0.021 (3)	−0.003 (2)	0.002 (2)	−0.001 (2)
C24	0.045 (3)	0.028 (3)	0.031 (3)	−0.006 (2)	0.019 (2)	0.003 (2)
C25	0.014 (2)	0.028 (3)	0.017 (3)	0.000 (2)	0.0006 (19)	−0.005 (2)
C26	0.012 (2)	0.024 (3)	0.019 (3)	−0.003 (2)	0.0029 (19)	0.002 (2)
C27	0.023 (2)	0.029 (3)	0.013 (3)	−0.002 (2)	0.004 (2)	−0.003 (2)
C28	0.021 (2)	0.026 (3)	0.019 (3)	−0.003 (2)	0.005 (2)	0.000 (2)
C29	0.038 (3)	0.027 (3)	0.027 (3)	−0.001 (2)	0.013 (2)	−0.002 (2)
C30	0.041 (3)	0.026 (3)	0.040 (3)	0.004 (2)	0.020 (3)	−0.004 (2)
C31	0.044 (3)	0.019 (3)	0.044 (4)	0.001 (2)	0.019 (3)	0.002 (2)
C32	0.032 (3)	0.031 (3)	0.033 (3)	−0.006 (2)	0.016 (2)	0.001 (3)
C33	0.017 (2)	0.022 (3)	0.020 (3)	−0.004 (2)	0.003 (2)	−0.001 (2)
C34	0.018 (2)	0.034 (3)	0.017 (3)	−0.005 (2)	−0.001 (2)	−0.001 (2)
C35	0.014 (2)	0.023 (3)	0.020 (3)	0.002 (2)	0.005 (2)	0.005 (2)
C36	0.014 (2)	0.026 (3)	0.024 (3)	0.003 (2)	0.006 (2)	0.001 (2)
C37	0.022 (2)	0.032 (3)	0.023 (3)	0.000 (2)	0.012 (2)	−0.003 (2)
C38	0.016 (2)	0.025 (3)	0.020 (3)	−0.002 (2)	0.000 (2)	−0.003 (2)

### Geometric parameters (Å, °)

**Table:**

O1—C2	1.223 (5)	C13—H13	0.9500
O2—C4	1.373 (5)	C14—C15	1.492 (6)
O2—C6	1.385 (4)	C15—C16	1.453 (6)
O3—C8	1.230 (5)	C16—C17	1.420 (6)
O4—C15	1.252 (5)	C17—C18	1.396 (5)
O5—C17	1.354 (5)	C18—C19	1.375 (5)
O5—H5	0.84 (1)	C18—H18	0.9500
O6—C21	1.231 (5)	C20—C21	1.501 (6)
O7—C25	1.385 (5)	C20—H20A	0.9800
O7—C23	1.395 (5)	C20—H20B	0.9800
O8—C27	1.224 (5)	C20—H20C	0.9800
O9—C34	1.248 (5)	C21—C22	1.464 (6)
O10—C36	1.352 (5)	C22—C23	1.375 (6)
O10—H10	0.84 (1)	C22—C38	1.456 (5)
C1—C2	1.502 (5)	C23—C24	1.461 (5)
C1—H1A	0.9800	C24—H24A	0.9800
C1—H1B	0.9800	C24—H24B	0.9800
C1—H1C	0.9800	C24—H24C	0.9800
C2—C3	1.469 (6)	C25—C26	1.368 (5)
C3—C4	1.361 (5)	C25—C38	1.410 (5)
C3—C19	1.453 (5)	C26—C35	1.424 (5)
C4—C5	1.493 (5)	C26—C27	1.496 (5)
C5—H5A	0.9800	C27—C28	1.488 (6)
C5—H5B	0.9800	C28—C29	1.385 (5)
C5—H5C	0.9800	C28—C33	1.403 (5)
C6—C7	1.374 (5)	C29—C30	1.388 (6)
C6—C19	1.407 (5)	C29—H29	0.9500
C7—C16	1.413 (5)	C30—C31	1.403 (6)
C7—C8	1.479 (6)	C30—H30	0.9500
C8—C9	1.487 (6)	C31—C32	1.383 (6)
C9—C10	1.390 (6)	C31—H31	0.9500
C9—C14	1.393 (5)	C32—C33	1.375 (6)
C10—C11	1.386 (6)	C32—H32	0.9500
C10—H10A	0.9500	C33—C34	1.490 (5)
C11—C12	1.397 (6)	C34—C35	1.447 (6)
C11—H11	0.9500	C35—C36	1.420 (5)
C12—C13	1.383 (6)	C36—C37	1.401 (6)
C12—H12	0.9500	C37—C38	1.380 (5)
C13—C14	1.407 (5)	C37—H37	0.9500
			
C4—O2—C6	106.6 (3)	C18—C19—C3	134.6 (4)
C17—O5—H5	104 (3)	C6—C19—C3	105.9 (4)
C25—O7—C23	106.9 (3)	C21—C20—H20A	109.5
C36—O10—H10	104 (4)	C21—C20—H20B	109.5
C2—C1—H1A	109.5	H20A—C20—H20B	109.5
C2—C1—H1B	109.5	C21—C20—H20C	109.5
H1A—C1—H1B	109.5	H20A—C20—H20C	109.5
C2—C1—H1C	109.5	H20B—C20—H20C	109.5
H1A—C1—H1C	109.5	O6—C21—C22	118.3 (4)
H1B—C1—H1C	109.5	O6—C21—C20	121.1 (4)
O1—C2—C3	119.6 (4)	C22—C21—C20	120.6 (4)
O1—C2—C1	121.0 (4)	C23—C22—C38	106.7 (4)
C3—C2—C1	119.4 (4)	C23—C22—C21	128.6 (4)
C4—C3—C19	105.8 (4)	C38—C22—C21	124.6 (4)
C4—C3—C2	130.3 (4)	C22—C23—O7	110.7 (4)
C19—C3—C2	123.9 (4)	C22—C23—C24	135.9 (4)
C3—C4—O2	112.2 (4)	O7—C23—C24	113.4 (4)
C3—C4—C5	136.2 (4)	C23—C24—H24A	109.5
O2—C4—C5	111.6 (4)	C23—C24—H24B	109.5
C4—C5—H5A	109.5	H24A—C24—H24B	109.5
C4—C5—H5B	109.5	C23—C24—H24C	109.5
H5A—C5—H5B	109.5	H24A—C24—H24C	109.5
C4—C5—H5C	109.5	H24B—C24—H24C	109.5
H5A—C5—H5C	109.5	C26—C25—O7	126.3 (4)
H5B—C5—H5C	109.5	C26—C25—C38	123.7 (4)
C7—C6—O2	126.4 (4)	O7—C25—C38	110.0 (4)
C7—C6—C19	124.1 (4)	C25—C26—C35	117.0 (4)
O2—C6—C19	109.4 (4)	C25—C26—C27	121.9 (4)
C6—C7—C16	116.5 (4)	C35—C26—C27	121.2 (4)
C6—C7—C8	122.5 (4)	O8—C27—C28	121.4 (4)
C16—C7—C8	121.1 (4)	O8—C27—C26	121.3 (4)
O3—C8—C7	121.5 (4)	C28—C27—C26	117.3 (4)
O3—C8—C9	120.7 (4)	C29—C28—C33	119.9 (4)
C7—C8—C9	117.8 (4)	C29—C28—C27	119.3 (4)
C10—C9—C14	119.9 (4)	C33—C28—C27	120.8 (4)
C10—C9—C8	119.6 (4)	C28—C29—C30	119.8 (4)
C14—C9—C8	120.5 (4)	C28—C29—H29	120.1
C11—C10—C9	120.5 (4)	C30—C29—H29	120.1
C11—C10—H10A	119.8	C29—C30—C31	120.0 (4)
C9—C10—H10A	119.8	C29—C30—H30	120.0
C10—C11—C12	119.8 (4)	C31—C30—H30	120.0
C10—C11—H11	120.1	C32—C31—C30	120.0 (4)
C12—C11—H11	120.1	C32—C31—H31	120.0
C13—C12—C11	120.3 (4)	C30—C31—H31	120.0
C13—C12—H12	119.9	C33—C32—C31	120.1 (4)
C11—C12—H12	119.9	C33—C32—H32	120.0
C12—C13—C14	119.8 (4)	C31—C32—H32	120.0
C12—C13—H13	120.1	C32—C33—C28	120.3 (4)
C14—C13—H13	120.1	C32—C33—C34	118.9 (4)
C9—C14—C13	119.7 (4)	C28—C33—C34	120.8 (4)
C9—C14—C15	120.6 (4)	O9—C34—C35	121.3 (4)
C13—C14—C15	119.7 (4)	O9—C34—C33	119.4 (4)
O4—C15—C16	121.6 (4)	C35—C34—C33	119.3 (4)
O4—C15—C14	119.3 (4)	C26—C35—C36	119.6 (4)
C16—C15—C14	119.0 (4)	C26—C35—C34	120.3 (4)
C7—C16—C17	119.9 (4)	C36—C35—C34	120.0 (4)
C7—C16—C15	120.1 (4)	O10—C36—C37	116.9 (4)
C17—C16—C15	120.0 (4)	O10—C36—C35	121.5 (4)
O5—C17—C18	115.6 (4)	C37—C36—C35	121.6 (4)
O5—C17—C16	122.8 (4)	C38—C37—C36	118.2 (4)
C18—C17—C16	121.6 (4)	C38—C37—H37	120.9
C19—C18—C17	118.5 (4)	C36—C37—H37	120.9
C19—C18—H18	120.8	C37—C38—C25	119.9 (4)
C17—C18—H18	120.8	C37—C38—C22	134.4 (4)
C18—C19—C6	119.5 (4)	C25—C38—C22	105.7 (3)
			
O1—C2—C3—C4	173.4 (4)	O6—C21—C22—C23	−176.5 (4)
C1—C2—C3—C4	−8.3 (7)	C20—C21—C22—C23	2.5 (7)
O1—C2—C3—C19	−4.8 (6)	O6—C21—C22—C38	0.8 (7)
C1—C2—C3—C19	173.4 (4)	C20—C21—C22—C38	179.8 (4)
C19—C3—C4—O2	−1.9 (5)	C38—C22—C23—O7	−1.3 (5)
C2—C3—C4—O2	179.6 (4)	C21—C22—C23—O7	176.4 (4)
C19—C3—C4—C5	177.4 (5)	C38—C22—C23—C24	178.7 (5)
C2—C3—C4—C5	−1.1 (8)	C21—C22—C23—C24	−3.6 (9)
C6—O2—C4—C3	1.0 (4)	C25—O7—C23—C22	0.5 (5)
C6—O2—C4—C5	−178.5 (3)	C25—O7—C23—C24	−179.5 (3)
C4—O2—C6—C7	179.6 (4)	C23—O7—C25—C26	178.8 (4)
C4—O2—C6—C19	0.4 (4)	C23—O7—C25—C38	0.6 (4)
O2—C6—C7—C16	−178.5 (4)	O7—C25—C26—C35	−176.4 (4)
C19—C6—C7—C16	0.5 (6)	C38—C25—C26—C35	1.7 (6)
O2—C6—C7—C8	1.4 (7)	O7—C25—C26—C27	2.4 (6)
C19—C6—C7—C8	−179.6 (4)	C38—C25—C26—C27	−179.5 (4)
C6—C7—C8—O3	8.5 (6)	C25—C26—C27—O8	4.7 (6)
C16—C7—C8—O3	−171.5 (4)	C35—C26—C27—O8	−176.5 (4)
C6—C7—C8—C9	−171.4 (4)	C25—C26—C27—C28	−175.1 (4)
C16—C7—C8—C9	8.5 (6)	C35—C26—C27—C28	3.7 (6)
O3—C8—C9—C10	−8.8 (6)	O8—C27—C28—C29	−3.1 (6)
C7—C8—C9—C10	171.2 (4)	C26—C27—C28—C29	176.7 (4)
O3—C8—C9—C14	173.8 (4)	O8—C27—C28—C33	176.1 (4)
C7—C8—C9—C14	−6.3 (6)	C26—C27—C28—C33	−4.1 (6)
C14—C9—C10—C11	0.9 (6)	C33—C28—C29—C30	1.7 (6)
C8—C9—C10—C11	−176.5 (4)	C27—C28—C29—C30	−179.1 (4)
C9—C10—C11—C12	−0.5 (7)	C28—C29—C30—C31	−2.0 (7)
C10—C11—C12—C13	−1.0 (7)	C29—C30—C31—C32	1.6 (7)
C11—C12—C13—C14	2.1 (7)	C30—C31—C32—C33	−0.9 (7)
C10—C9—C14—C13	0.2 (6)	C31—C32—C33—C28	0.6 (7)
C8—C9—C14—C13	177.6 (4)	C31—C32—C33—C34	179.7 (4)
C10—C9—C14—C15	−179.3 (4)	C29—C28—C33—C32	−1.0 (6)
C8—C9—C14—C15	−1.8 (6)	C27—C28—C33—C32	179.8 (4)
C12—C13—C14—C9	−1.7 (6)	C29—C28—C33—C34	179.9 (4)
C12—C13—C14—C15	177.7 (4)	C27—C28—C33—C34	0.7 (6)
C9—C14—C15—O4	−173.1 (4)	C32—C33—C34—O9	2.9 (6)
C13—C14—C15—O4	7.5 (6)	C28—C33—C34—O9	−178.1 (4)
C9—C14—C15—C16	8.0 (6)	C32—C33—C34—C35	−175.8 (4)
C13—C14—C15—C16	−171.4 (4)	C28—C33—C34—C35	3.3 (6)
C6—C7—C16—C17	−1.8 (6)	C25—C26—C35—C36	−1.0 (6)
C8—C7—C16—C17	178.3 (4)	C27—C26—C35—C36	−179.8 (4)
C6—C7—C16—C15	177.5 (4)	C25—C26—C35—C34	179.0 (4)
C8—C7—C16—C15	−2.4 (6)	C27—C26—C35—C34	0.2 (6)
O4—C15—C16—C7	175.3 (4)	O9—C34—C35—C26	177.7 (4)
C14—C15—C16—C7	−5.8 (6)	C33—C34—C35—C26	−3.7 (6)
O4—C15—C16—C17	−5.4 (7)	O9—C34—C35—C36	−2.3 (6)
C14—C15—C16—C17	173.5 (4)	C33—C34—C35—C36	176.3 (4)
C7—C16—C17—O5	−178.6 (4)	C26—C35—C36—O10	178.0 (4)
C15—C16—C17—O5	2.1 (6)	C34—C35—C36—O10	−2.0 (6)
C7—C16—C17—C18	1.9 (6)	C26—C35—C36—C37	0.0 (6)
C15—C16—C17—C18	−177.5 (4)	C34—C35—C36—C37	180.0 (4)
O5—C17—C18—C19	180.0 (3)	O10—C36—C37—C38	−177.7 (4)
C16—C17—C18—C19	−0.5 (6)	C35—C36—C37—C38	0.3 (6)
C17—C18—C19—C6	−0.9 (6)	C36—C37—C38—C25	0.3 (6)
C17—C18—C19—C3	−178.7 (4)	C36—C37—C38—C22	177.9 (4)
C7—C6—C19—C18	0.9 (6)	C26—C25—C38—C37	−1.3 (6)
O2—C6—C19—C18	−179.9 (3)	O7—C25—C38—C37	177.0 (4)
C7—C6—C19—C3	179.3 (4)	C26—C25—C38—C22	−179.6 (4)
O2—C6—C19—C3	−1.5 (5)	O7—C25—C38—C22	−1.3 (4)
C4—C3—C19—C18	−179.9 (4)	C23—C22—C38—C37	−176.4 (5)
C2—C3—C19—C18	−1.3 (7)	C21—C22—C38—C37	5.9 (7)
C4—C3—C19—C6	2.1 (5)	C23—C22—C38—C25	1.5 (4)
C2—C3—C19—C6	−179.3 (4)	C21—C22—C38—C25	−176.2 (4)

### Hydrogen-bond geometry (Å, °)

**Table:**

*D*—H···*A*	*D*—H	H···*A*	*D*···*A*	*D*—H···*A*
O5—H5···O4	0.84 (1)	1.82 (2)	2.596 (4)	153 (4)
O10—H10···O9	0.84 (1)	1.77 (3)	2.552 (4)	153 (6)
